# ‘This Is Real Misery’: Experiences of Women Denied Legal Abortion in Tunisia

**DOI:** 10.1371/journal.pone.0145338

**Published:** 2015-12-18

**Authors:** Selma Hajri, Sarah Raifman, Caitlin Gerdts, Sarah Baum, Diana Greene Foster

**Affiliations:** 1 Groupe Tawhida Ben Cheikh, Tunis, Tunisia; 2 Advancing New Standards in Reproductive Health (ANSIRH), Bixby Center for Global Reproductive Health, University of California San Francisco (UCSF), Oakland, California, United States of America; 3 Ibis Reproductive Health, Oakland, California, United States of America; London School of Economics, UNITED KINGDOM

## Abstract

Barriers to accessing legal abortion services in Tunisia are increasing, despite a liberal abortion law, and women are often denied wanted legal abortion services. In this paper, we seek to explore the reasons for abortion denial and whether these reasons had a legal or medical basis. We also identify barriers women faced in accessing abortion and make recommendations for improved access to quality abortion care. We recruited women immediately after they had been turned away from legal abortion services at two facilities in Tunis, Tunisia. Thirteen women consented to participate in qualitative interviews two months after they were turned away from the facility. Women were denied abortion care on the day they were recruited due to three main reasons: gestational age, health conditions, and logistical barriers. Nine women ultimately terminated their pregnancies at another facility, and four women carried to term. None of the women attempted illegal abortion services or self-induction. Further research is needed in order to assess abortion denial from the perspective of providers and medical staff.

## Introduction

Unsafe abortion continues to pose a significant health risk to women around the world leading to persistent high rates of maternal mortality and morbidity [1]. Unsafe abortion is the second leading cause of maternal mortality worldwide [2]. More than one-fifth of all pregnancies in 2008 ended in abortion and half of those (49%) were unsafe, up from 44% in 1995 [3]. Most unsafe abortions occur in developing countries [3]. Women depend on access to safe abortion to postpone or stop childbearing, for socioeconomic concerns (including disruption of education or employment and resource constraints for existing children), relationship problems, risks to maternal or child health, and pregnancy resulting from rape or incest [4]. Safe abortion access is an essential component of comprehensive sexual and reproductive health care, as defined by the International Conference on Population and Development (ICPD) Programme of Action [5] and the World Health Organization [6].

Although legalization of abortion has improved the availability of safe abortion in many places, such as South Africa [7] and Nepal [8], legalization alone is insufficient. Barriers to accessing legal care can perpetuate high rates of unsafe and extralegal abortion. These barriers include lack of awareness about abortion laws, lack of financial resources, geographical obstacles, lack of access to medications, requirements of unnecessary approvals or tests, coercion, and stigma [9],[10]. Beyond legalization, ensuring access to safe abortion for all women requires comprehensive medical training [11–14]; inclusion of multiple types of providers in abortion provision [15–18]; establishment and enforcement of health and safety standards and resources [19]; information and referrals [20], [21]; and affordable quality care [22].

### Tunisia

Tunisia has long been at the forefront of providing legal access to safe and affordable reproductive health services for women [23]. Though Muslims constitute more than 99% of the population [24], the government has supported a uniquely secular position on reproductive health services. In 1965, Tunisia became the first Muslim country to liberalize its abortion law, making the service legal for women who had more than five children, were within the first trimester, and had approval from their husbands [25]. The Tunisian government further liberalized the law in 1973 to make abortion legal for *any* woman within the first trimester regardless of husband’s approval [26]. Tunisia was the first African country to legalize mifepristone for medication abortion up to nine weeks in 2000 [27]. Since 2001, the use of medical abortion has become increasingly common in Tunisia; it is selected by more than 70 percent of women seeking abortion [28] and has recently become available to women in public facilities.

Significant demographic change has occurred in Tunisia in the last 20 years, following efforts to increase access to reproductive health services, including abortion. Total fertility fell from 3.13 in 1990–95 to 2.05 in 2005–10 [29]. Maternal mortality rates declined significantly, from 68.9 per 100,000 births in 1993–94 to 36.3 per 100,000 births in 2005–2007 [30]. Enrollment of girls in secondary school rose from 28% to 94% and women’s labor force representation increased from 20.8% to 25.3% despite overall rises in unemployment between 1990 and 2010 [31].

Abortion is legal upon request in the first trimester by a physician legally practicing in a hospital, health-care establishment or authorized clinic since 1974 by law and with the creation of the National Family Planning Office (ONFP). After the first trimester, abortion is permitted in approved establishments to preserve physical and mental health of the mother or in the case of fetal anomaly; in these cases the treating physician must present a report to the physician who will perform the abortion. Women who seek abortion for mental health reasons must go through a psychiatric assessment and receive a certificate from a specialist. The government subsidizes medication and surgical abortion services; all women seeking first trimester abortion can obtain it free of charge in public hospitals and family planning clinics [26].

Most health care in Tunisia is provided through the public system, which is structured in to three tiers: primary health care is provided by more than 2200 primary health centers and 108 district hospitals; secondary care is provided by 32 regional hospitals; and tertiary care is provided by a network of 29 academic hospitals. Ten percent of all hospital beds are in the private sector [32]. Physicians are able to provide abortion in all facilities licensed by the state (public or private) within the constraints of the law. In the public sector, only university hospitals provide second trimester abortion. Private sector abortion services are expensive and vary by gestational age, estimated by providers and clinics using recommendations for surgical procedures in Tunisia. The cost of abortion in the private sector often constitutes a significant portion of an average monthly income in Tunisia.

A lack of data and peer-reviewed literature makes it difficult to evaluate recent changes in abortion access in Tunisia. Advocates on the ground, however, report a deterioration in access to abortion in recent years, which may stem from government budget cuts (beginning in 2004) and increasing political conservatism since the Arab Spring in 2010–2011 [33]. Beginning in the 1980s, 24 family planning clinics, one in each governorate, were authorized to provide free abortion services, but not all of the facilities actually did so due to shortages in resources and training. After the legalization of medication abortion in 2001, ten of the clinics replaced their surgical abortion services with medication abortion services. While this increased access to medication abortion, the availability of surgical abortion declined: only five family planning centers still offer surgical abortion [33]. The 2008 global economic crisis and growing conservatism resulted in the end of abortion provision altogether at some clinics in the more conservative and rural north west region, where poverty is highest in the country and employment and literacy are low, and in the southern region, which is mostly semi-arid desert [34]. Outside medical contexts, abortion remains a taboo subject and women lack adequate information about the availability of legal services; as a result, informal sector abortion with traditional medicine or drugs from a pharmacy, continues to be practiced [26]. A previous study has shown that approximately one-quarter of women are denied wanted abortions in the country’s capital: 7 percent were turned away for gestational age reasons, 15 percent were required by the clinic to undergo unnecessary laboratory tests, and 4 percent were either required by the clinic to have an ultrasound scan prior to the procedure or were referred to other providers [35]. Anecdotal accounts indicate that some physicians in Tunisia are evoking conscientious objection for religious reasons to refuse to perform abortion in hospitals and in some private facilities and that this has become more common since the 2010–2011 revolution. Some of these providers give advice and referrals to women while others do not. As in many other places [11], conscientious objection is poorly understood with regard to the law and circumstances in which providers can invoke their right to refuse to provide.

Due to a lack of data and persistent stigma surrounding the issue, the reasons why women are denied wanted legal abortions and the actions they take following denial are not well documented in Tunisia. This study seeks to understand the abortion experiences of women who are denied care in Tunisia, including reasons for seeking abortion, barriers faced accessing abortion services, reasons for and experiences after being turned away and pregnancy outcomes. This study is part of a multi-country Global Turnaway Study, which recruited women at the moment they were denied services from one or two facilities (often one public and one private facility) in five different countries [35]. Researchers in each country followed up with all eligible women who consented to participate two months after recruitment to conduct in-depth interviews with each participant. This standardized model of recruitment and data collection was implemented in Tunisia, Nepal [9], Bangladesh, South Africa (10), and Colombia. Open-ended interview guides enabled interviewers to explore country-specific issues as they arose [35].

## Methods

The study was conducted in Tunis, the capital of Tunisia, located in the northern region, over a two-month period in 2013. Women were recruited from one public and one private facility to capture experiences in both sectors. Recruitment clinics were selected based on willingness to participate as a study site and client volume; the public hospital registry indicated a rate of 1,000 abortions per year, the highest in the country. Both of the initial study sites recruited in Tunis agreed to participate. Two researchers approached all women who presented for abortion at the recruitment facility, with approval from the facility, and asked whether they would participate in the study. If the potential participant expressed interest, the researcher led her to a private location at the clinic and gave her materials about the study. The recruiters obtained informed consent, followed women throughout their visit to determine whether or not they obtained an abortion that day, and conducted a brief questionnaire to ensure eligibility for the study. Women were eligible to participate in the study if they were 18–49 years old, seeking abortion services, and denied wanted services that day for any reason. Eligible women were interviewed two months later. The two month time-frame allowed researchers to learn about women’s experiences after being turned away; it was necessary to provide women time to experience the denial and decide on their next course of action. Researchers obtained verbal consent at the time of recruitment and again at the time of interview, in addition to audio-recording consent. Given the sensitivity of the study, written consent was not obtained to maintain confidentiality. Contact details were obtained only after women agreed to participate in the study. The interviewer sent text-messages or phone call reminders prior to the scheduled interview. In-depth interviews with women were conducted by a female Tunisian psychologist, bilingual in Tunisian Arabic and French and previously trained in conducting qualitative interviews. She has prior experience conducting interviews with women in urban and rural areas, as well as conducting values clarification trainings for providers on contraception and abortion.

Participation in the study consisted of a semi-structured in-depth interview two months after denial. The interview guide was open-ended and included suggested probes. The key topics explored included: reasons for seeking abortion; reasons for denial and subsequent responses; the decision-making process after being denied; knowledge of abortion law and facilities where abortion is provided; knowledge of unlicensed providers and self-induction methods; and recommendations for other women. Copies of the research instrument are available upon request. Ethical approval was granted by Le Comite d’Ethique du Service A du CMNT in Tunis, Tunisia as well as the Committee on Human Research at the University of California, San Francisco (IRB #10–04511). Phone numbers and names were collected, but participants were assured that they would not be identified by name, facility or any other identifier in all forms of dissemination, including publications and at meetings. Data were stored in password protected computer files.

Interview transcripts were translated for analysis from Tunisian Arabic to French and then from French to English, due to the Arabic translator’s better knowledge of French. Tunisian Arabic is a specific Mahgrebi dialect, which is different from Standard or Classical Arabic and spoken by far fewer people; this limited our ability to find a translator both fluent in Tunisian Arabic and English. Funding constraints inhibited hiring a researcher to analyze the data in Tunisian Arabic. There are challenges to cross-language qualitative research [36]; in order to cope with these challenges, the research team was careful to communicate frequently with the Tunisia-based team to confirm specific meanings of words and ensure that the translation accurately captured original meanings.

The data were analyzed using a thematic approach. The primary coder, trained in qualitative research methods, conducted the primary analysis, generating initial codes and documenting emerging themes. After additional iterations of the codebook were complete, in consultation with the co-authors, the codes were applied to all interviews using Dedoose 5.0.11 (SocioCultural Research Consultants: Los Angeles, CA). Relevant socio-demographic data was identified in the transcripts and synthesized in an Excel spreadsheet. Key themes and illustrative quotations were reviewed for consensus with regard to interpretation by the entire research team. The following information is provided in parenthesis with each quotation in this paper: an identification number, self-reported gestational age at the time the participant first sought abortion services, and the participant’s pregnancy outcome. Facility names have been extracted and replaced with generic names which are consistent across respondents’ stories, such as “Hospital 1.”

## Results

Twenty-two women were recruited. Nine women (41%) were lost to follow up, as a result of unanswered phone calls from research staff or refusal to participate without providing specific reasons. Thirteen women participated in semi-structured in-depth interviews approximately two months after denial. Eleven interviews were conducted by telephone and two were conducted in person in a private space. The interviews lasted for an average of 26 minutes (range: 14 to 40 minutes). Two women, who were interviewed by phone, ended their interviews prematurely. Neither woman could be reached again. These unfinished interviews were nevertheless included in the analysis due to the richness of the data that was available.

### Participant characteristics and overview pregnancy outcomes

Basic socio-demographic data are presented in Table 1. The mean age of the women interviewed was 30 years old, with a range from 22–43 years. Nine of the women were married with at least one child. The women in the sample had an average of two children. Four women were single or engaged and nulliparous. Five women indicated that they were employed outside of the home. Though women were not asked directly about contraceptive use, four women indicated that they had been using oral contraception before they became pregnant and four women indicated that they had not been using any form of contraception. The remainder did not comment on use of contraception and were not probed further by the interviewer.

**Table 1:** Socio-demographic data and pregnancy outcomes.

ID	Age	Gestational Age at ultrasound	Marital Status	Children	Pregnancy Outcome	Abortion Method	Abortion location
ID1	Missing	16 weeks	Married	4	Carried to term	N/A	N/A
ID8	27	20 weeks	Single	0	Carried to term	N/A	N/A
ID12	30	1 week	Married	4	Carried to term	N/A	N/A
ID6	30	16 weeks	Married	4	Carried to term	N/A	N/A
ID2	37	8 weeks	Married	3	Abortion	Surgical	Private clinic
ID3	43	4 weeks	Widow	3	Abortion	Medication	Hospital
ID4	Missing	5 weeks	Married	2	Abortion	Medication	Hospital
ID5	22	Unknown	Engaged	0	Abortion	Medication and surgical	Hospital and private clinic
ID7	32	8 weeks	Married	2	Abortion	Medication	Private clinic
ID9	23	6 weeks	Engaged	0	Abortion	Surgical	Private clinic
ID10	22	9 weeks	Engaged	0	Abortion	Surgical	Private clinic
ID11	34	3 weeks	Married	3	Abortion	Methotrexate and medication abortion	Hospital
ID13	30	6 weeks	Married	1	Abortion	Medication and surgical	Hospital and private clinic

Among the 13 women interviewed, nine women terminated their pregnancies and the remaining four women carried their pregnancies to term. Among women who had an abortion, one woman received a shot of methotrexate due to a suspected ectopic pregnancy in addition to medication abortion, three received surgical curettage, three received medication abortion, and two received both surgical and medication abortion. Six out of nine women obtained their abortions at private facilities. All three women who were in the second trimester when they were denied abortion services ultimately carried their pregnancies to term, even though two of these women sought abortion for health reasons and therefore should have been eligible for second trimester abortion services.

### Decision and experience seeking abortion care

#### Pregnancy recognition

The majority of women recognized their pregnancies during the first trimester, including all the women who ultimately terminated their pregnancies and two of the four women who carried to term. When asked how they recognized that they were pregnant, some women explained that they experienced delays in menstruation and/or common signs of pregnancy, such as dizziness and exhaustion. Two women found out they were pregnant in the second trimester. One woman reported that she believed her symptoms were related to an illness rather than pregnancy: ‘I only found out when I fainted. So I went for some tests thinking that it was perhaps diabetes, since there is a history of this disease in my family. Then I decided to do a [pregnancy test], which turned out positive’ (ID8, 20 weeks, carried to term). The other woman who was in her fourth month when she recognized the pregnancy explained that her menstruation was irregular, sometimes with delays up to 28 weeks, and that she took a test only when she started feeling tired and dizzy (ID1, 16 weeks, carried to term). Five women reported using pharmacy-bought pregnancy tests, available to women without prescription in pharmacies but at a cost, before seeking abortion services at a facility. Pregnancy tests are more accessible to women who are able to pay for them, thereby potentially delaying pregnancy recognition and consequently abortion-seeking for many women.

#### Reasons for seeking an abortion

Across the sample, one of the primary reasons reported for seeking abortion was maternal health concern. Three out of four of the women who ultimately carried to term and three out of nine who terminated their pregnancies sought abortion for health reasons. Health concerns varied, from fainting, dizziness, and anemia, thrombosis and blood clots, diabetes and large organ failure.

Several others reasons for abortion were also mentioned, including lack of financial resources, birth spacing, advanced age, and being unmarried. Some women explained that they did not want any more children, due to the physical, financial, social and psychological demands required. One woman concerned that having a child would prohibit her from being able to work and meet financial requirements: ‘You have no idea how difficult it is to rent. It is very, very expensive. It is impossible! My husband cannot pay for everything alone, and as I told you, I cannot put my daughter in baby daycare to be able to go and work. I have to wait until she is one year old’ (ID13, 6 weeks, medication and surgical abortion). Another woman explained that she already had young children and wanted to space her births: ‘And me pregnant, taking care of my 10-month-old daughter, how would I have managed? When I am fainting? Should my husband take a leave from work? Should my mother leave her work?’ (ID13, 6 weeks, medication and surgical abortion).

All four women who identified as single and nulliparous said their relationship status was part of the reason for seeking abortion. One of these women was pressured to seek abortion by her fiancé because they were not married, even though she wanted to continue the pregnancy: 'Yes, he was always saying “you have to have an abortion, you have to have an abortion” so that I would go ahead. I was saying “no” and he was saying “yes”. In the end this created some problems in our relationship, and finally I gave in to avoid any escalation’ (ID8, 20 weeks, carried to term). Another woman talked about the consequences she felt she deserved: ‘I did not want to have a child without being married. I accepted all the punishment and my responsibilities, and I swore to myself that I would never [have sex again] except after being married’ (ID10, 9 weeks, surgical abortion).

### Experiences with denial

Three main categories of denial experiences emerged from the interviews with women in this study: denial due to gestational age, denial due to health conditions of the woman, and denial due to imposition of substantial logistical and bureaucratic barriers. This third category is considered a form of denial because it was unlikely that women who faced these barriers would have ever obtained their wanted abortions had they stayed at the facility. Many women obtained referrals to other providers or facilities on the day they were denied abortion, particularly those women who were denied due to advanced gestational age or health concerns. In some cases, women were denied services at multiple facilities and due to a combination of the above three reasons.

#### Denied for gestational age

Women denied for gestational age were turned away for being too early and too far along in pregnancy. One woman was told she was too early for medical abortion, one woman reported denial both for being too early and, later in the pregnancy, for being too far along, and two other women were denied from public facilities because they were beyond the facility gestational limit.

One woman who was denied medical abortion for being too early in her pregnancy sought abortion at three weeks gestation and explained: ‘…they did not go ahead at first, because they told me that the fetus had to grow a bit more before they could give me pills’ (ID11, 3 weeks, methotrexate and medication abortion). She was later diagnosed with and treated for an ectopic pregnancy.

The other woman who sought services early in her first trimester, reportedly at one week, was originally told to return later: ‘In fact when I was already one week pregnant, she told me that the fetus was too small for a sonogram…I responded that I had a blood test which was positive. She told me to come to see her in one month. They made me come and go until the fetus was big enough’ (ID12, 1 week, carried to term). She later returned for a sonogram and was then referred from one public hospital to another, until she was ultimately sent back to the original facility where she sought care. There, she was denied abortion again due to advanced gestational age, even though she believed that she was only six weeks pregnant at the time and that the provider was inaccurately reading the ultrasound: ‘When I went the last time, she did a sonogram and she lied telling me that I was at the third month. I responded that the previous time she had told me that I was one week pregnant and now I am three months pregnant, and how could that happen so fast? [The provider said] “He’s developed, it’s a sin”‘ (ID12, 1 week, carried to term).

Neither this woman, nor the other two women who were turned away for being over the gestational limits were aware of their legal right to access abortion after 12 weeks for mental and physical health reasons and only one of them was evaluated for a second trimester abortion according to the physical and mental health exceptions. This woman, whose husband pressured her to have an abortion despite her desire to carry the pregnancy to term, received a psychiatric assessment at 20 weeks gestation but did not receive a certificate for abortion.

High costs and lack of partner support prevented two women from obtaining care at the private clinic where they had been referred after being denied services for being beyond the gestational limit at a public facility. The woman who was denied during her process for both early and later gestation was asked whether she tried to go to a private clinic she replied: ‘No, I would have liked to. I talked to my husband about it, but he refused, and asked me to keep the baby. He is the one who told me to keep the baby. He said he could spend two hundred dinars for me, but “Imagine how god will punish you after”‘ (ID12, 1 week, carried to term). Another woman also describes trying to get financial support from her husband: ‘He promised to give me money for an abortion and now that it is the fourth month, I will not be able to have an abortion. He was just making fun of me’ (ID6, 16 weeks, carried to term). The third woman did seek private care after being denied at a public facility only to then be intimidated and persuaded by the private doctor to not have the abortion after all: ‘When I went to the doctor’s he told me that I was taking a bigger risk in aborting the pregnancy than in staying pregnant, meaning that if I stayed pregnant I would be under control but if I had an abortion I would risk a hemorrhage and I would become sick. In brief, I kept the baby because I was at risk and that’s it’ (ID1, 16 weeks, carried to term).

#### Denied for health conditions

Women were also denied services and referred for various health reasons. Women reported being referred to other facilities because they were diabetic, had asthma, and had previously taken an anticoagulant medication for blood clotting. Women expressed confusion about why their health conditions were impacting their access to abortion care. The woman who was referred from one hospital to another because she was asthmatic believed her condition had nothing to do with her abortion.

[*At Hospital 4] they told me that since I had asthma*, *I had to go to Hospital 2[…]*. *I told her that I wasn’t ill*, *and that she should prescribe the abortion*. *[…] She told me that I had to go to Hospital 2 for an abortion and that here [at Hospital 4] they no longer performed abortions*. *When I told her I wasn’t ill*, *and that I was sure of my decision*, *she told me to go to Hospital 2*, *and that they performed abortion there’ (ID13*, *6 weeks*, *medication and surgical abortion)*.

Later when she went to Hospital 2, she was told that they did not perform abortions (‘There at the counter, I saw a woman who told me that they did not do abortions there…Directly! She did not even ask me why or how!’) and was then sent to another hospital, where she was told she would have to wait two months (‘Same thing there, the midwife said: “Now, no. We will do it in a month or two.”‘).

The woman who was denied care because of potential blood clotting explained that her denial was based on the fact that she had taken an anticoagulant medication during her previous pregnancy. But since she was no longer taking the medication (a provider told her that she no longer needs it), she was confused as to why this would preclude her from getting an abortion:

I: In your opinion, why did they turn you down on an abortion?

*Honestly*, *I do not know*. *Maybe because previously*, *with my youngest son*, *I was taking Sintrom*. *She told me that because I was taking Sintrom*, *they could not do an abortion*.

I: And why are you taking Sintrom?

*Because during my previous pregnancy one of the arteries in my lungs became obstructed*. *…I took it for two years*, *or perhaps one year and a half*. *Then they saw that I was doing better*, *and they told me to stop completely*.

I: But now that you are pregnant, you are not taking Sintrom.

*No*, *now luckily*, *now I am doing fine*. *(ID12*, *1 week*, *carried to term)*

In some cases, women sought abortion due to concerns about health conditions, but instead the clinic used those conditions as a reason to deny care. For example, one woman, aged 43 years old, sought abortion because of her diabetes and her advanced age but was then denied care because she was diabetic: ‘The doctor denied the abortion. She said it was because I was diabetic and that I had to go to the hospital. But [at the hospital], they sent me back because my glucose levels were not very high… [even though] the doctor had told me that if she did it, it would be complicated because of the diabetes’ (ID3, 4 weeks, medication abortion). In other cases, women were not given the opportunity to explain their health concerns to their providers. One woman explained why she believed she was not physically able to have another child: ‘My body will not be able to cope. I have anemia, and sometimes I get dizzy. My situation is hard… I am exhausted at the highest level!’ (ID6, 16 weeks, carried to term). But when she went to a hospital for care, they denied her services immediately without asking why she wanted an abortion and referred her to a private clinic instead (due to her advanced gestational age), where she was unable to afford the cost of services. Another woman, who was left with no choice but to continue the pregnancy, sought abortion because she was anemic and had thrombosis but was denied at a public hospital without being asked why she wanted an abortion: ‘[At the hospital] she told me that we were not having an abortion, without asking me any questions, she told me we weren’t having an abortion because the child was already developed’ (ID1, 14 weeks, carried to term). In contrast, one woman who did have the opportunity to explain her health concerns to her provider received very different treatment: ‘I told [the provider] that I was ill, and you know the conditions of my life and all, and my health. I consulted him, and he said that if you are ill, you are obliged to have an abortion, and the doctor who was monitoring my health [at another facility] said the same thing. She told me to have an abortion’ (ID4, 5 weeks, medication abortion).

#### Denied for logistical and bureaucratic delays

Several women were not explicitly denied care but instead told to wait long periods or to go home and return later for care. This was considered a denial of care because women were not able to get the care they wanted on that day and they reported it was unlikely that they would ever have successfully obtained an abortion at the given facility. Some women were made to wait for many hours: ‘To begin with at the hospital, you have to find a doctor […] we spent the whole day there. We went from 8 am to 5 pm and in the end the doctor did not come, and sometimes, when you are waiting in line, you go for a snack, and then come back, and you cannot find the doctor and you cannot say anything’ (ID7, 8 weeks, medication abortion). Many women described being referred back and forth between departments at a hospital or between facilities: ‘…as you know they send you from one service department to another, and this one will not handle your case, nor the other. We really had a hard time then’ (ID11, 3 weeks, methotrexate and medication abortion). Other women were told to return on another day, sometimes weeks in the future; for example: ‘You have to wait more than a week. It does not matter to them that this is an emergency … They take their time…Can you imagine waiting 3 days for a test, and 3 more days for a sonogram, and 4 days to see the doctor… If you add up the days you will see that the whole month has gone by…’ (ID9, 6 weeks, surgical abortion).

Many of the women who faced delays in public facilities left and went to private clinics because they were worried the delays would limit their ability to have an abortion. One woman was sent back and forth between providers at Hospital 2 for five days, during which time she said it was difficult to leave her children at home alone. She explained: ‘It was already my second month, and when I went to see them they gave me an appointment for a sonogram in two weeks. And I thought that in the interim the fetus would …grow too much. So I went to a private practice doctor’ (ID2, 8 weeks, surgical abortion). Another woman returned to the same hospital eight times and then decided to seek care at a private clinic: ‘I don’t remember when I went there, but I know that I went there eight times, and that this took too much time, and so I went to a private clinic…’ (ID5, unknown gestation, medication and surgical abortion). Two women refused to wait for services at a hospital after they realized that the doctors had gone on strike; instead they went to private clinics where they obtained surgical abortions:

*At Hospital 1 they are slow…they said either that the doctors were on strike*, *or that the test results would only be ready in three to six days*, *or this*, *and that…so that it meant everything would take a lot of time*, *do you understand*? *[…] And this didn’t work for me considering that I was already one and half months pregnant*. *It was an emergency*, *and I was not going to wait*. *(ID9*, *6 weeks*, *surgical abortion)*

*[…] I went for the test and the sonogram but the doctor was not there because they were on strike*. *I understood that this meant more delay*. *I borrowed money from someone and I went for an abortion at a [private] clinic for 250 DT (~$150)*. *(ID10*, *9 weeks*, *surgical abortion)*

Women were frustrated that they could not hold the providers and facilities responsible for delaying their care and potentially making it more difficult if not impossible to receive an abortion. One woman who had an incomplete medication abortion following care at a hospital endured bleeding for a month and a half until she could receive a surgical procedure at a private facility to complete the abortion. She visited two health centers and then two hospitals before she gave up on the public sector due to delays and explained how the referral system failed her:

*‘The problem is that they do not give any written papers*, *no proof which could harm them… Normally*, *Hospital 4 should have given me a paper*. *They did not give me one… they do not give you any proof and just leave you as is… if I wanted to file a complaint*, *I do not have any papers… There was no supervision*. *If there were supervision for everything*, *then things would not happen this way’ (ID13*, *6 weeks*, *medication and surgical abortion)*.

### Private versus public services

Those women who were able to obtain care at private facilities commented on the higher quality care available in private clinics compared to public facilities. One woman recalls her experience at a private clinic, compared to a public hospital: ‘It lasted a total of 4 hours, and I left without any harm, thank God. They took good care of me, and they really helped me … it was a lot easier than at the hospital, and the people over there were not telling all sorts of stories. […] If you had a “situation”, I would tell you that if you had money and you could pay for everything, it is better for you to go to a [private] clinic’ (ID9, 6 weeks, surgical abortion). Another woman commented on the culture of anxiety that exists at public facilities, as compared to private clinics: ‘[At the hospital], all the patients are in the same place waiting for the doctor, without knowing whether she is coming or not. There is so much quarreling and fear! […] It is horror, and you know that you will not come back. Everyone is very nervous’ (ID7, 8 weeks, medication abortion). Public hospital providers were seen as more judgmental about a woman’s decision to have an abortion. For example, one woman said: ‘They tortured me by asking me all these “Why?” questions. And [they said] “it’s a sin”… as you know! Yes, at the hospital, they drive you crazy, with lots of questions, why this and that, and why did you make a mistake, and why are you not protected…’ (ID2, 8 weeks, surgical abortion). Several women said that they would recommend to other women in need of abortion services to go to private clinic services if they can: ‘Those who have the means should go [to a private clinic] directly. They will find cleanliness and purity there’ (ID13, 6 weeks, medication and surgical abortion).

### Partner involvement and impact on abortion experience

Partner influence did seem to play a role in women’s ability to ultimately obtain an abortion, even after denial. The women who ultimately terminated their pregnancies either had support from their partners (‘My husband… always says that I should do what suits me best, on condition only that I inform him’ (ID13, 6 weeks, medication and surgical abortion)) or chose not to disclose the pregnancy and abortion-seeking process to their partners for fear of their disapproval. Those in the latter category were often unmarried or were having conflicts with their husband. The women who ultimately terminated their pregnancies were steadfast in their decision, even when they were encouraged by friends or acquaintances to continue the pregnancy. One woman said, ‘Honestly, everyone had their own opinion. But … no one could really influence my decision. That’s all. It was my decision (ID11, 3 weeks, methotrexate and medication abortion). Another woman explained: ‘They tried in my neighborhood…”keep it, let it grow,” but I knew that I shouldn’t, that it would make me sick, and prevent me from working…’ (ID13, 6 weeks, medication and surgical abortion).

Three out of the four women who ultimately did not terminate the pregnancy faced opposition from their partners regarding terminating the pregnancy, particularly after they had been turned away at least once. Though these women were initially able to either convince their partners or seek abortion services on their own, their partners’ discouragement became increasing difficult to oppose after they were denied care. One woman explains how she was able to convince her husband to support her and then how defeated they both began to feel when she faced numerous logistical barriers over two months seeking abortion in the public healthcare system:

*Truth be told*, *my husband did not want an abortion*. *When he found out that I was pregnant*, *he asked me to keep the child*, *but I resisted*. *I told him that he would not be taking care of the child with me*. *And that I would be spending sleepless nights alone*, *and to let me have the abortion*. *He was convinced*, *and told me to do what I wanted to do*. *As I was going from one hospital to another*, *he also gave up*, *and told me to let it be with all the coming and going*, *and to keep it*, *and just stop running around*. *(ID12*, *1 week*, *carried to term)*

Among the women who carried to term, the only woman who received support from her partner was single; her partner initially told her to get an abortion because they were not married and when she was turned away from one facility he supported her decision to carry to term.

### Impact of abortion denial

Across the board, women were dissatisfied with the care they received throughout the abortion seeking process, including women who ultimately carried their pregnancies to term as well as women who successfully received wanted abortions (due to the process required in order to do so). One woman who continued her pregnancy explained the significant impact the pregnancy has had on her family since she was denied care: ‘Four children, and a fifth one on top! Where are we heading this way? Even his family is talking about it… Poverty and tyranny. I do not know what to do anymore. I do not even have clothes for the newborn. This is real misery’ (ID6, carried to term). Another woman who visited at least three different public facilities, some more than once, explained: ‘In sum, during this period of time, they really fooled me’ (ID12, 1 week, carried to term).

The women who ultimately terminated their pregnancies reflected on how difficult the process was and how the delays and referrals were discouraging, exhausting and frustrating. One woman said: ‘In sum, everything was really chaos. They really interfered with our lives and made us feel depressed’ (ID11, 3 weeks, Methotrexate and medication abortion). Another woman described how she felt after being denied services at a public facility after multiple referrals: ‘I suffered…when they turned me away, I no longer had any hope’ (ID13, 6 weeks, medication and surgical abortion).

## Discussion

The women interviewed for this study faced significant challenges to accessing safe and legal abortion services, including personal barriers such as lack of partner support or financial means to seek abortion quickly, as well as logistical and systematic barriers, including ineffective referral systems and service delays. All of the women interviewed visited at least two, and in many cases three or more, different facilities before either deciding to carry the pregnancy to term or finally receiving abortion services.

Women were turned away from abortion care for three main reasons: gestational age (early or advanced), medical contraindications, and logistical barriers and delays. Those who were beyond three months gestation were denied an abortion on request (in accordance with the abortion law) but also never given the opportunity to be assessed for a second trimester abortion in the context of their physical or mental health (not in accordance with the abortion law) [26]. It is difficult to assess whether women were referred appropriately for medical contraindications. There is a lack of protocol in Tunisia about how and where women with chronic medical conditions should access abortion care. According to the literature that is available, it is unclear whether abortion-related mortality in the first trimester is more likely to occur in women with chronic medical problems [37]. Yet, there is evidence to suggest that women with serious medical problems, including diabetes and hypertension, face an increased risk of adverse health events during pregnancy, including increased pregnancy-related morbidity and mortality [38, 39]. Prompt abortion care, for those who want it, reduces these health risks, because their condition may deteriorate further with advanced pregnancy. Referral from lower level clinics to hospitals may be required if a patient requires surgical abortion, for example, and the clinic is not adequately equipped.

The only woman who may have required surgical abortion, because she had taken anticoagulant medication and therefore has a higher risk of post-abortion hemorrhage with medication abortion drugs [40], was referred between hospitals rather than from a clinic to a hospital. It remains unclear whether or not she was actually at increased risk of hemorrhage given that she was no longer on anticoagulant medication. The other two health conditions for which women were referred are not considered as contraindications to abortion in the U.S. context, according to a study by Guiahi and Davis [41]; yet, without clinical records or confirmation from providers, whether or not referral in this context was warranted is unclear. These cases in particular beg the question of whether some providers are objecting to provide abortion care, for whatever reason, either overtly or covertly.

It is not surprising that many women endured service delays at public facilities, given that public expenditure on the health sector in Tunisia has slowed since the 1990s and public health services have experienced declines in quality and availability (31). Whether service delays were due to inefficiency, provider conscientious objection, stigma or even malevolence of specific providers and medical staff is unclear [42, 43]. Regardless of the cause, these service delays negatively affected women’s quality of care experiences and postponed a procedure that is well known to be safer early on in pregnancy [44]. Women who recognized this and were able to gather the funds sought care in a private clinic, but those that could not afford it were left with no other choice but to continue waiting. Whether or not the women who obtained abortions in private clinics would have been able to do so had they waited for care in the public system is unknown.

As anticipated with an exploratory qualitative study, the sample is not representative of all women in Tunisia. Women in our sample are likely wealthier, more educated and more urban than the average woman in Tunisia. And our results do not include the experiences of women under 18 years of age or the experiences of women who seek abortion outside facility-based care. Despite this, the data provide a deeper understanding of women’s experiences seeking abortion in a country where little data is available.

Additionally, the data provide information from only the women’s perspective, rather than both the women and the providers and facility staff. As a result, it is difficult to determine the root causes of denial and whether or not denial or referral was medically indicated. It is possible that women were rightfully referred for services elsewhere; it is also possible, for example, that providers were unavailable, inadequately trained, or objected to providing abortion on moral and religious grounds. The authors plan next to interview providers to better understand provider motivations.

Our findings suggest that opportunities exist at the moment of denial of abortion to improve access to safe abortion services in Tunisia. Currently, minimal support and information is provided to women at the moment they are turned away from a facility. Our findings demonstrate a clear misinterpretation of the abortion law in Tunisia, attributable to either malevolent providers or inadequate provider training, or both. Improved provider training, including values clarification [45], and community awareness-building initiatives could improve knowledge and accountability from both sides. Enhanced referral systems, which simultaneously equip women with the knowledge they need to successfully seek a legal abortion, such as that in Colombia where a clinic-based advocacy group counsels all women who are denied services, could reduce unnecessary delays and risks to women’s health. This study provides new and important insight on the experiences of women seeking legal abortion services in Tunisia. These findings suggest a need for greater understanding of provider experiences with abortion denial as well as systematic, quantitative data collection to better understand the health and socioeconomic consequences of legal abortion, illegal abortion and childbirth.

## Supporting Information

S1 Text Interview Guide. (DOCX)

## References

[1] Grimes DA, Benson J, Singh S, Romero M, Ganatra B, Okonofua FE, et al Unsafe abortion: the preventable pandemic. Lancet. 2006;368(9550):1908–19. 10.1016/S0140-6736(06)69481-6 . 17126724

[2] Say L, Chou D, Gemmill A, Tuncalp O, Moller AB, Daniels J, et al Global causes of maternal death: a WHO systematic analysis. Lancet Glob Health. 2014;2(6):e323–33. Epub 2014/08/12. 10.1016/S2214-109X(14)70227-X . 25103301

[3] Sedgh G, Singh S, Shah IH, Ahman E, Henshaw SK, Bankole A. Induced abortion: incidence and trends worldwide from 1995 to 2008. Lancet. 2012;379(9816):625–32. 10.1016/S0140-6736(11)61786-8 . 22264435

[4] Bankole AS, S.; Haas T. Reasons why women have induced abortions: evidence from 27 countries International Family Planning Perspectives. 1998;24(3). doi: http://www.guttmacher.org/pubs/journals/2411798.html.

[5] UN. Report of the international conference on population and development: Programme of Action adopted at the International Conference on Population and Development, Cairo. Cairo: United Nations; 1995.

[6] WHO. Sexual and reproductive health: core competencies in primary care In: Organization WH, editor. Geneva 2011.

[7] Jewkes R, Rees H, Dickson K, Brown H, Levin J. The impact of age on the epidemiology of incomplete abortions in South Africa after legislative change. Bjog. 2005;112(3):355–9. Epub 2005/02/17. 10.1111/j.1471-0528.2004.00422.x . 15713153

[8] Henderson JT, Puri M, Blum M, Harper CC, Rana A, Gurung G, et al Effects of abortion legalization in Nepal, 2001–2010. PLoS One. 2013;8(5):e64775 Epub 2013/06/07. 10.1371/journal.pone.0064775 23741391 PMC3669364

[9] Puri MV, D.; Gerdts C.; Foster D.G. "I need to terminate this pregnancy at any cost": Effect of being denied abortion on women's lives in Nepal. International perspectives on sexual and reproductive health. 2015 10.1186/s12905-015-0241-y PMC4606998 26466784

[10] Harries J, Gerdts C, Momberg M, Greene Foster D. An exploratory study of what happens to women who are denied abortions in Cape Town, South Africa. Reproductive health. 2015;12(1):21 Epub 2015/04/18. 10.1186/s12978-015-0014-y 25884479 PMC4371847

[11] Harries J, Cooper D, Strebel A, Colvin CJ. Conscientious objection and its impact on abortion service provision in South Africa: a qualitative study. Reproductive health. 2014;11(1):16 10.1186/1742-4755-11-16 24571633 PMC3996040

[12] Rehnstrom Loi U, Gemzell-Danielsson K, Faxelid E, Klingberg-Allvin M. Health care providers' perceptions of and attitudes towards induced abortions in sub-Saharan Africa and Southeast Asia: a systematic literature review of qualitative and quantitative data. BMC Public Health. 2015;15(1):139 Epub 2015/04/18. 10.1186/s12889-015-1502-2 25886459 PMC4335425

[13] Tey NP, Yew SY, Low WY, Su'ut L, Renjhen P, Huang MS, et al Medical students' attitudes toward abortion education: Malaysian perspective. PLoS One. 2012;7(12):e52116 10.1371/journal.pone.0052116 23300600 PMC3531402

[14] Berer M. Making abortions safe: a matter of good public health policy and practice. Bull World Health Organ. 2000;78(5):580–92. Epub 2000/06/22. 10859852 PMC2560758

[15] Sneeringer RK, Billings DL, Ganatra B, Baird TL. Roles of pharmacists in expanding access to safe and effective medical abortion in developing countries: a review of the literature. J Public Health Policy. 2012;33(2):218–29. 10.1057/jphp.2012.11 22402571 PMC3510770

[16] Paul M, Gemzell-Danielsson K, Kiggundu C, Namugenyi R, Klingberg-Allvin M. Barriers and facilitators in the provision of post-abortion care at district level in central Uganda—a qualitative study focusing on task sharing between physicians and midwives. Bmc Health Services Research. 2014;14. doi: Artn 28 10.1186/1472-6963-14-28 . PMC3903434 24447321

[17] Kopp Kallner H, Gomperts R, Salomonsson E, Johansson M, Marions L, Gemzell-Danielsson K. The efficacy, safety and acceptability of medical termination of pregnancy provided by standard care by doctors or by nurse-midwives: a randomised controlled equivalence trial. Bjog. 2015;122(4):510–7. Epub 2014/07/22. 10.1111/1471-0528.12982 . 25040643

[18] Ngoc NT, Shochet T, Blum J, Hai PT, Dung DL, Nhan TT, et al Results from a study using misoprostol for management of incomplete abortion in Vietnamese hospitals: implications for task shifting. BMC Pregnancy Childbirth. 2013;13:118 Epub 2013/05/24. 10.1186/1471-2393-13-118 23697561 PMC3704810

[19] Rogo KO, Aloo-Obunga C, Ombaka C, Oguttu M, Orero S, Oyoo C, et al Maternal mortality in Kenya: the state of health facilities in a rural district. East Afr Med J. 2001;78(9):468–72. . 11921579 10.4314/eamj.v78i9.8977

[20] Banerjee SK, Andersen KL, Baird TL, Ganatra B, Batra S, Warvadekar J. Evaluation of a multi-pronged intervention to improve access to safe abortion care in two districts in Jharkhand. BMC Health Serv Res. 2014;14:227 10.1186/1472-6963-14-227 24886273 PMC4035795

[21] Janiak E. Fostering Abortion Referrals in the Healthcare and Social Service Settings: Lessons from the Literature and from Key Informant Interviews. Cambridge: 2012.

[22] Ilboudo PG, Somda SM, Sundby J. Key determinants of induced abortion in women seeking postabortion care in hospital facilities in Ouagadougou, Burkina Faso. Int J Womens Health. 2014;6:565–72. 10.2147/IJWH.S60709 24920938 PMC4045174

[23] Nazer I. The Tunisian experience in legal abortion. Int J Gynaecol Obstet. 1980;17(5):488–92. . 6103849 10.1002/j.1879-3479.1980.tb00196.x

[24] Pew Forum on Religion & Public Life. Mapping the global Muslim population: a report on the size and distribution of the world's Muslim population Washington, District of Columbia: Pew Research Center; 2009. 59 pages p.

[25] MOH. Tunisian Action Plan. In: Health TMo, editor. Tunis 1991.

[26] UN. Abortion Policies A Global Review. United Nations Population Division 2002.

[27] Blum J, Hajri S, Chelli H, Mansour FB, Gueddana N, Winikoff B. The medical abortion experiences of married and unmarried women in Tunis, Tunisia. Contraception. 2004;69(1):63–9. Epub 2004/01/15. . 14720623 10.1016/j.contraception.2003.08.019

[28] Hajri S, Blum J, Gueddana N, Saadi H, Maazoun L, Chelli H, et al Expanding medical abortion in Tunisia: women's experiences from a multi-site expansion study. Contraception. 2004;70(6):487–91. 10.1016/j.contraception.2004.06.012 . 15541411

[29] United Nations DoEaSA, Population Division. World Population Prospect: The 2012 Revision. United Nations, 2013.

[30] Farhat EB, Chaouch M, Chelli H, Gara MF, Boukraa N, Garbouj M, et al Reduced maternal mortality in Tunisia and voluntary commitment to gender-related concerns. International journal of gynaecology and obstetrics: the official organ of the International Federation of Gynaecology and Obstetrics. 2012;116(2):165–8. Epub 2011/11/22. 10.1016/j.ijgo.2011.10.010 . 22098789

[31] Boughzala MH, M.T. Promoting Inclusive Growth in Arab Countries: Rural and Regional Development and Inquality in Tunisia. Working Paper No71 Global Economy and Development2014.

[32] WHO. WHO-AIMS Report on Mental Health System in Tunisia. 2008. doi: http://www.who.int/mental_health/tunisia_who_aims_report.pdf.

[33] Naili H. Tunisia Loses Ground on Safe Affordable Abortion: Womensenews.org; 2012 [updated 12 December 2012; cited 15 April 2015]. News website. Available: http://womensenews.org/story/abortion/121211/tunisia-loses-ground-safe-affordable-abortion#.VCycDfldVHU.

[34] Ohier F. Abortion in Tunisia: A Shifting Landscape: www.tunisia-live.net; 2014 [updated 27July 2014; cited 15 April 2015]. News website. Available: http://www.tunisia-live.net/2014/07/27/abortion-in-tunisia-a-shifting-landscape/

[35] Gerdts C, DePiñeres T, Hajri S, Harries J, Hossain A, Puri M, et al Denial of abortion in legal settings. J Fam Plann Reprod Health Care. 2014 10.1136/jfprhc-2014-100999 . 25511805 PMC4501171

[36] Squires A. Methodological challenges in cross-language qualitative research: a research review. International journal of nursing studies. 2009;46(2):277–87. 10.1016/j.ijnurstu.2008.08.006 18789799 PMC2784094

[37] Guiahi M, Davis A. First-trimester abortion in women with medical conditions: release date October 2012 SFP guideline #20122. Contraception. 2012;86(6):622–30. 10.1016/j.contraception.2012.09.001 23039921

[38] Curtis K. U.S. Medical Eligibility Criteria for Contraceptive Use, 2010: Adapted from the World Health Organization Medical Eligibility Criteria for Contraceptive Use, 4th edition In: Division of Reproductive Health NCfCDPaHP, editor. Centers for Disease Control 2010 p. 1–6.

[39] Chang J, Elam-Evans LD, Berg CJ, Herndon J, Flowers L, Seed KA, et al Pregnancy-related mortality surveillance—United States, 1991–1999. MMWR Surveill Summ. 2003;52(2):1–8. . 12825542

[40] Davey A. Mifepristone and prostaglandin for termination of pregnancy: contraindications for use, reasons and rationale. Contraception. 2006;74(1):16–20. 10.1016/j.contraception.2006.03.003 . 16781254

[41] Guiahi M, Davis A, Planning SoF. First-trimester abortion in women with medical conditions: release date October 2012 SFP guideline #20122. Contraception. 2012;86(6):622–30. 10.1016/j.contraception.2012.09.001 . 23039921

[42] Harries J, Orner P, Gabriel M, Mitchell E. Delays in seeking an abortion until the second trimester: a qualitative study in South Africa. Reprod Health. 2007;4:7 10.1186/1742-4755-4-7 17883835 PMC2039724

[43] van Bogaert LJ. The limits of conscientious objection to abortion in the developing world. Dev World Bioeth. 2002;2(2):131–43. . 12870481 10.1111/1471-8847.00046

[44] Drey EA, Foster DG, Jackson RA, Lee SJ, Cardenas LH, Darney PD. Risk factors associated with presenting for abortion in the second trimester. Obstet Gynecol. 2006;107(1):128–35. 10.1097/01.AOG.0000189095.32382.d0 . 16394050

[45] Turner KL, Hyman AG, Gabriel MC. Clarifying values and transforming attitudes to improve access to second trimester abortion. Reproductive health matters. 2008;16(31 Suppl):108–16. Epub 2008/09/17. 10.1016/S0968-8080(08)31389-5 . 18772091

