# Supplementary material for: ‘This Is Real Misery’: Experiences of Women Denied Legal Abortion in Tunisia
Source: PLoS One. 2015 Dec 18;10(12):e0145338. doi: 10.1371/journal.pone.0145338 (PMC4686168; doi:10.1371/journal.pone.0145338)
Supplement: S1 Text — (DOCX) [file pone.0145338.s001.docx]

**Global Turn Away Study**

**UCSF**

**2013**

Guideline for In-depth interviews with women

Introduction

- Introduce yourself and thank the participant for agreeing to meet you.
- Explain the purpose of the study and how the interview will be conducted (why s/he was selected, recording, what the information will be used for).
- Before you start the interview, obtain written informed consent.

**Key reminders for Interviewer**

- The participant was selected because she was denied to receive service from the place she first visited.
- The aim of the interview is to better understand:
- Experiences seeking abortion services at various places.
- Decision making process to end the pregnancy and choice of places
- Impact on personal life and family wellbeing and future plan.
- The interview guide is only a guide. Try to cover all of the issues but there is considerable flexibility for the participant to tell you what s/he feels is important, and for you to find out about other interesting/related issues.
- Each question is followed by possible probes and issues to cover. You should only use these as examples or to generate ideas, not as a checklist.
- The ordering of the questions is intentional but you don’t have to follow it if the respondent begins discussing something of interest which hasn’t yet been covered; just return to the earlier question afterwards.
- Try to build trust and rapport with the respondent throughout the interview.
- Probe where necessary and clarify vague or contradictory information. Use prompts like:
  - Can you tell me more about that?
  - What do you mean exactly?
  - Am I right in thinking that…?
  - What do you think about…?
  - Do you mean that…?
  - Earlier you told me…but here you seem to be saying…Can you clarify this?

**1. Participant Background**

| **Topic Focus** | **Core questions** | **Additional questions or prompts** |
| --- | --- | --- |
| **Personal Background** | - Could you please tell me about yourself? | - Age, age at marriage, number of living children, (number of son and daughter, if just one whether son or daughter) ethnicity, place of living, socio-economic status of family, occupation, level of education, partners’ education and occupation, whether or not currently living with partner |

**2. Decision making process**

| **Decision making** | - I would like you to tell me what was happening in your life at the time of this pregnancy? - Why was the pregnancy considered unintended? - Do you recall the circumstances that urged you to think in that direction (advice for abortion)? | - When did you first discover that you were pregnant? How did feel about it at that time? - What was your partner’s initial reaction to this pregnancy? - What circumstances initially compelled you to decide to terminate the pregnancy? - Do you remember talking to anyone before discussing the decision to go for an abortion? If so, who did you talk to about the pregnancy? What advice were you looking for? What information did you receive? - Was the decision to abort influenced by the advice given by others you may have spoken with? If so, who influenced your decision? - Was there anyone else involved in this discussion about terminating the pregnancy? - Who had the final say on the decision? - What considerations did you have to think about in making this decision? - Were there any difficulties (impediments) that you faced in making this decision? - How long did it take from the initial decision making to actually visiting a provider? - What factors do you think kept you from coming to the clinic earlier in your pregnancy |
| --- | --- | --- |

**3. Abortion seeking behavior**

| **Experiences with the clinic visit** | | | - Could you please tell me what happened at the clinic/hospital when you went for an abortion | | - How did you feel about what happened? - What were some of the reasons you weren’t able to get an abortion at [clinic name] that day? - Did you have an ultrasound test before coming to this facility? | |
| --- | --- | --- | --- | --- | --- | --- |
| **Subsequent attempt for abortion** | What did you do after you left the clinic?  Did you ever consider getting an abortion elsewhere/going to another clinic/provider?  If yes, can you tell me about this? *( please ask about all the process and all places visited)*  Why did you or did you not seek an abortion elsewhere? | | - Who do you talk to? - Who, if anyone, did you seek advice from about what to do next? - Do you remember talking to anyone? From where? From whom? What information advice were you given? | |  |  |
|  | ***If sought abortion elsewhere***  Tell me what happened when you went to this provider. [*try to capture the chronology of events, concerns or dilemmas that were occurring, and what emotions were involved]*  Was the abortion successful? If no, why not? | | - - - Without giving me anyone’s name, what kind of person did you seek an abortion from/where did you go?     - What happened?     - How did you feel about what happened? (*probe to see if she felt it was a safe place/if she felt safe, probe to see if she is aware about government approved place*)     - How much did the abortion cost?     - Were you satisfied with the outcome? What concerns do you remember having? Did you get any advice about seeking medical care after the aobrtion? What was the advice?     - Did you experience any complications following the abortion?   If so, what kind of complications did you experience?   - - - Did you seek medical or other care after the abortion? What medical care did you seek and why?     - Is there anything you wish that you had known before going to this provider? | |  |  |
| **Self tried for abortion** | ***If not visited elsewhere for an abortion:***  Did you ever consider ending the pregnancy yourself?  If yes, can you tell me about that thought process?  Did you try to end the pregnancy yourself? Why or why not?  **If tried by herself:**  How did you try to end the pregnancy yourself?  **If used medicines (both effective or ineffective) :**  Can you tell me about your experience trying to end the pregnancy yourself?  Was the abortion successful?  **If used other traditional methods:**  Can you tell me about your experience trying to end the pregnancy yourself?  Was the abortion successful? | | - Did you seek any information or advice about it? - From where? From whom? What information advice you were given? (*ask separately for each source of advice*) - What happened? - How did you feel about what happened? - Without giving me anyone’s name, where did you get the drugs? - How much did it cost? - What, if any, directions did you get about using the drugs? - Without giving me anyone’s name, where did you get directions about using the drug?   - - Did you experience any complications following the abortion?   If so, what kind of complications did you experience?   - Did you seek medical or other care after the abortion? - What medical care did you seek and why? - What, if any, advice or information did you get about ending the pregnancy yourself? - What happened? - How did you feel about what happened?   - - Did you experience any complications following the abortion?   If so, what kind of complications did you experience?   - Did you seek medical or other care after the abortion? What medical care did you seek and why? | |  |  |

**4. Future plan and impact on family life and wellbeing**

| **Impact** | **Ask if woman is still pregnant**  What are your plans for after the baby is born? | - How has your relationship with your family changed now that you are having a baby? - How has your relationship with your partner changed now that you are having a baby? - How do you think that having a baby will affect your life? |
| --- | --- | --- |

**5. Knowledge about the laws and services**

| **Knowledge about the law and services** | Tell me what you know about the abortion law in Tunisia?  Do you remember when you and your partner decided to terminate the pregnancy, were either of you aware about the abortion law in our country? | - Have you heard anything about the new abortion law? On what conditions a woman can have legal abortion in Tunisia? *(Be sure to note carefully any misinformation or incorrect information respondent might have about the existing law)* |
| --- | --- | --- |
|  | Of the women you know, how many might have terminated a pregnancy at some time or the other? *(Probe: Think of your friends, relatives, and neighbors)* | - Without telling me their names, how do you know they may have terminated a pregnancy? How well do you know them? |
|  | Around here, if a woman could not get a legal abortion and wanted to terminate a pregnancy, do you think it would be easy or difficult for her to do it? Why? |  |
|  | If a woman could not get a legal abortion, can you tell me what are some ways that people say a woman could terminate a pregnancy? |  |
|  | If a woman could not get a legal abortion, are there places or people a woman could go if she wanted to terminate a pregnancy? Where? To whom? |  |
| **Advice for others seeking abortion** | If someone approached you about an unintended pregnancy, someone who is experiencing the same situation that you had and they asked you what they should do, what would you say to them? Why? |  |
